# Physiotherapy informed by Acceptance and Commitment Therapy for chronic low back pain: A mixed‐methods treatment fidelity evaluation

**DOI:** 10.1111/bjhp.12583

**Published:** 2022-02-03

**Authors:** Melissa N. Galea Holmes, Vari Wileman, Shaira Hassan, Julie Denning, Duncan Critchley, Sam Norton, Lance M. McCracken, Emma Godfrey

**Affiliations:** ^1^ 4616 Health Psychology Section Department of Psychology Institute of Psychiatry, Psychology and Neuroscience (IoPPN) King's College London UK; ^2^ 4616 Department of Physiotherapy School of Population Health and Environmental Sciences Faculty of Life Sciences and Medicine King's College London UK; ^3^ Present address: Department of Applied Health Research University College London UK; ^4^ Present address: Department of Psychology Uppsala University Sweden

**Keywords:** fidelity assessment, chronic low back pain, physical therapy, acceptance and commitment therapy, randomized controlled trial

## Abstract

**Objectives:**

A randomized controlled trial of a new type of Physiotherapy informed by Acceptance and Commitment Therapy (PACT), found that it improved functioning in people with chronic low back pain compared to usual physiotherapy care. Fidelity evaluation is necessary to understand trial processes and outcomes. This study evaluated PACT treatment fidelity including delivery, receipt, and enactment.

**Design:**

A mixed‐methods study nested within a randomized controlled trial was conducted.

**Methods:**

A total of 72 (20% of total) PACT treatment audio files were independently assessed by two raters, according to a novel framework developed to measure PACT treatment content adherence, therapeutic alliance, ACT competence, and treatment enactment. Interview transcripts from 19 trial participants randomized to PACT were analysed thematically for evidence of treatment receipt and enactment.

**Results:**

PACT physiotherapists delivered treatment as intended with high content adherence and satisfactory therapeutic alliance, but ACT competence was low. Qualitative findings indicated participant receipt of 11/17 and enactment of 3/17 components; 89% (*n* = 17) and 47% (*n* = 9) of participants reported treatment receipt and enactment of at least one component, respectively.

**Conclusions:**

This mixed‐methods study of PACT treatment demonstrated high fidelity reflecting treatment content delivery and receipt, and therapeutic alliance. There was some evidence of treatment enactment in participants with chronic low back pain. Low ACT competence could be addressed through additional support and adaptations to therapeutic processes for delivery by physiotherapists.

**Statement of contribution**

***What is already known on this subject?***

- Psychologically informed physiotherapy is a recommended treatment for chronic low back pain.
- Comprehensive treatment fidelity evaluations should address multiple domains, including delivery and engagement.
- In an efficacy trial, physiotherapy informed by acceptance and commitment therapy improved functioning in people with chronic low back pain compared with usual care

***What does this study add?***

- Physiotherapists adhered to treatment content delivery and achieved a therapeutic alliance
- Therapeutic competence was low suggesting a need for further training in or adaptation of underlying processes
- Treatment receipt was reported, particularly for patient guide use, mindfulness, and identifying SMARTER goals

## Background

Chronic low back pain is a complex, multifactorial condition that is associated with disability and psychological morbidity (Demyttenaere et al., 2007; Hartvigsen et al., 2018; Hoy et al., 2012). Physiotherapy is frequently combined with psychological treatment in comprehensive pain management programmes. More recently, the integration of psychological approaches within physiotherapy practice itself has gained popularity. Psychologically informed physiotherapy acknowledges the need to assess and manage patient cognitions and emotional responses regarding their condition, as well as physical symptoms and functioning (Keefe, Main, & George, 2018). Incorporating psychological approaches within their clinical practice might be challenging for some physiotherapists and reaching a consensus on appropriate methods and effective training is required. Potential implementation challenges are also recognized including restructuring of existing outpatient services (Coronado et al., 2020). Process evaluations of psychologically informed physiotherapy trials contribute to this important developing area, highlighting opportunities for refinement. This paper reports on a process evaluation from the Physiotherapy informed by Acceptance and Commitment Therapy (PACT) Study (Godfrey et al., 2019).

The PACT intervention is novel, underpinned by the psychological flexibility model, which describes the capacity to persist in or modify behaviour such that an individual is open to experience, connected to the present moment, and engaged in actions linked to valued goals (Feliu‐Soler et al., 2018; McCracken & Morley, 2014). In this context, people are encouraged to focus on improving function rather than reducing pain. In a multicentre randomized controlled trial, PACT reduced disability and improved functioning compared with usual care physiotherapy (appropriate individual or group treatment as standard in the [UK National Health Service]) in people with chronic low back pain at the end of treatment (Godfrey et al., 2016, 2019). This is consistent with broader evidence that psychological interventions delivered by physiotherapists demonstrate small improvements in pain, disability, and depression compared with usual care physiotherapy (Guerrero, Maujean, Campbell, & Sterling, 2018).

While these findings are promising, understanding how or why psychologically informed physiotherapy is effective and how to enhance and optimize roll‐out of interventions such as PACT relies on evidence of treatment fidelity; that is, the extent to which an intervention is implemented as intended (Bellg et al., 2004; Borrelli, 2011; Borrelli et al., 2005). Treatment fidelity supports the reliability and validity of intervention and ensures outcomes can be attributed to proposed components and processes, not variability in implementation.

The Behaviour Change Consortium identified five domains of fidelity including study design, training, delivery, receipt, and enactment (Bellg et al., 2004; Borrelli, 2011; Borrelli et al., 2005). This framework informed the PACT study design and the training programme provided to physiotherapists delivering the intervention, which are reported elsewhere (Galea Holmes et al., 2020; Godfrey et al., 2016, 2019). The present study evaluates fidelity of delivery, receipt, and enactment. Delivery encompasses provider competency (i.e., the extent to which a provider achieved and maintained the skills targeted during training) and adherence (i.e., the extent to which treatment components are delivered as intended). These are distinct constructs and independent predictors of treatment outcomes (Cross & West, 2011). Participant receipt (i.e., participant understanding and ability to use the skills and recommendations provided during treatment) and enactment (i.e., participant ability to apply learning to relevant real‐life settings) can be described collectively as engagement (Walton, Spector, Williamson, Tombor, & Michie, 2020).

In addition, non‐specific factors that may influence delivery, such as therapeutic alliance, should be assessed to understand their impact on treatment outcomes (Borrelli et al., 2005). Therapeutic alliance contributes to positive outcomes of physiotherapy, including treatment adherence, mood, physical functioning, and satisfaction with care (Hall, Ferreira, Maher, Latimer, & Ferreira, 2010; Moore, Holden, Foster, & Jinks, 2020). Patients who received psychologically informed physiotherapy for severe chronic pain distinguished the therapeutic relationship from their previous treatment experiences and reported authentic interactions that transcended patient‐clinician roles and a biopsychosocial approach that considered the whole person as factors central to behaviour change (Wilson, Chaloner, Osborn, & Gauntlett‐Gilbert, 2017).

While guidance exists on approaches to treatment fidelity evaluation (Bellg et al., 2004; Borrelli et al., 2005; Toomey et al., 2020; Walton et al., 2020), persistent challenges include the need for reliable, valid, and feasible measures that are relevant to bespoke interventions, and limited resourcing of time and expertise required to conduct the evaluation. In addition, few studies of behavioural interventions evaluate delivery, receipt, and enactment to provide a comprehensive understanding of treatment fidelity (Rixon et al., 2016; Walton, Spector, Tombor, & Michie, 2017). In a review of 22 studies describing physiotherapist‐led group‐based self‐management interventions for chronic low back pain and osteoarthritis, Toomey, Currie‐Murphy, Matthews, and Hurley (2015) found overall poor adherence to validated recommendations for implementing and assessing treatment fidelity, with only 20%, 33%, and 43% of components present that reflected delivery, receipt, and enactment, respectively. However, the review only evaluated the reporting of strategies to enhance implementation fidelity, and there remains a need to evaluate fidelity outcomes to determine the extent to which those strategies achieved their aims.

The aim of this study was to conduct a process evaluation of the PACT intervention fidelity, including treatment delivery, engagement, and therapeutic alliance. We designed a fidelity evaluation that was feasible to conduct within the context of a randomized controlled trial, which incorporated a tailored checklist, established measures, and qualitative evidence. We assessed audio‐recorded PACT treatment sessions for evidence of treatment delivery, therapeutic competence, treatment receipt, and enactment. Further, we evaluated interviews with people with chronic low back pain who received PACT for further evidence of treatment receipt and enactment.

## Methods

### The PACT study

The PACT Study compared PACT with usual care physiotherapy. A total of 248 (*n* = 124 randomized to PACT) participants with chronic low back pain were recruited from physiotherapy clinics in four [UK] public hospitals [Guy’s Hospital, St Thomas’ Hospital, King’s College Hospital, and Ashford & St Peter’s Hospital]. The PACT intervention consisted of two individual 60‐min face‐to‐face treatment sessions (sessions 1 and 2) and one 20‐min remote telephone treatment session (session 3) delivered by specially trained physiotherapists (*n* = 8). The training was delivered by a clinical psychologist, health psychologist, and physiotherapist and included a treatment manual, 2 days of face‐to‐face group learning, at least two individual supervision sessions while practicing PACT delivery, individual written or oral feedback on up to two audio‐recorded PACT treatment sessions, plus ongoing monthly group supervision. Details of the intervention and training programme were reported previously (Galea Holmes et al., 2020; Godfrey et al., 2016, 2019).

Ethical approval was granted by the National Research Ethics Committee South Central – Berkshire; 14/SC/0277). Consent to audio record and analyse PACT treatment sessions was provided by participants prior to data collection. The study was conducted in accordance with the Data Protection Act 1998 and with the Data Protection Policy of [King’s College London (UK)].

### Sampling

#### Sampling of audio‐recorded PACT treatment sessions

A random sample of 72 audio‐recorded PACT treatment sessions was included in the analyses. This sample reflected 20% of the expected dataset of 360 total sessions delivered as outlined in the trial protocol (Godfrey et al., 2016), which is the optimal fidelity sample percentage (Borrelli, 2011). Sampling was conducted by a statistician and was stratified by the physiotherapist and then session number to ensure adequate physiotherapist and site variation was achieved, and to account for the potential learning effects resulting from ongoing supervision provided to physiotherapists over the duration of the trial (Plumb & Vilardaga, 2010). The stratified random sampling balanced the number of sessions selected across physiotherapists. As it is reasonable to expect that those physiotherapists delivering more sessions would do so with greater fidelity, this sampling method was likely to result in conservative estimates of fidelity. This sampling also resulted in an imbalance in the overall number selected per session because not all sessions were delivered by all physiotherapists (e.g., due to participant dropout, missed attendance). In addition, some audio recordings were missing or inaudible and could not be included. Therefore, the final sample included *n* = 32, 27, and 13 samples of sessions 1, 2, and 3, which relates to 36%, 42%, and 36% of the total available audio recordings for each session, respectively.

#### Qualitative interview sampling

Participants were individuals with chronic low back pain who received the PACT intervention as part of a randomized controlled trial (Godfrey et al., 2016, 2019). A pragmatic, purposive sampling approach was conducted, which aimed to recruit approximately 20% of PACT recipients to achieve a dataset that was sufficient and feasible to analyse (Borrelli, 2011; Braun & Clarke, 2012). The sampling method ensured participants were invited who represented all trial sites and treating physiotherapists, and who reflected the demographic variation within the intervention group (age, gender, hospital site, and baseline disability measured using the Roland Morris Disability questionnaire (RMDQ; Roland & Morris, 1983)). In total, 24 individuals were invited to participate following their 3‐month follow‐up assessment (trial primary outcome measure endpoint): three did not respond and two did not attend, leaving a final sample of 19 individuals included in the study (mean age: 59 years (*SD* = 13.1), *n* = 11 female, mean baseline disability (RMDQ) score: 13.8 [*SD* = 5.4]).

### PACT intervention fidelity measures

#### PACT intervention content

The PACT intervention included 17 key theoretically defined components (Table 1) that physiotherapists had been trained to deliver. A measure to assess adherence to PACT content delivery was developed to mirror a treatment checklist used by physiotherapists to support and supplement the delivery of each session. The PACT content measure included 10, 6, and 6 items assessed in sessions 1, 2, and 3, respectively. Among these, 20 items were rated to reflect adherence to delivery as: ‘not completed’, ‘partially completed,’ or ‘completed’. Two items were scored on a categorical scale (yes and no) to assess whether a PACT patient guide was provided, and a handshake or verbal agreement was exchanged (i.e., a behavioural technique representing a commitment to agreed goals; both session 1). A percentage of adherence (not completed/partially completed/completed) was calculated for each item.

**Table 1:** PACT treatment content and fidelity analysis results indicating proportions of sampled sessions for which content was delivered completely

		Not completed (%)	Partially completed (%)	Completed (%)
Session 1				
Sets the agenda	Outlines structure, schedule, and delivery of treatment; establish the foundation for a good therapeutic alliance	6	28	66
Conducts brief physical assessment	Identify and rule out red flags	3	0	97
Covers feedback	Explains that no serious medical problems have been uncovered and that it is safe to gradually resume activities	0	0	100
Shifts focus from pain to function	Rather than struggling with pain, suggests openness to another approach and presents the goal of PACT, to help people function better especially in the areas that are important to them	3	3	94
Helps patient identify SMARTER goals:	(1) Engages patient in identifying core values and setting related goals	0	6	94
	(2) breaks goals down into small steps	0	3	97
	(3) records agreed goals in the patient manual	0	0	100
Addresses barriers to goal attainment	Encourages patients to consider and prepare for potential barriers to goal fulfilment. Implements strategies to promote openness, awareness, and engagement, e.g., mindfulness exercises and action plans in response to potential barriers	3	3	94
Teaches ‘notice five things’ mindfulness exercise^a^	Physiotherapist demonstrates ‘notice five things’ and reinforces how the patient can use this skill anytime on their own to help when they are struggling with their pain	3	0	97
Uses at least one metaphor or tool^b^	At any point during the session, at least one metaphor or tool should be referred to	3	3	94
Provides PACT patient guide^c^	Giving the patient knowledge to use outside of the sessions and after treatment	0	n/a	100
Handshake or verbal agreement^c^	Making a public commitment to agreed goals	3	n/a	97
Session 2				
Responds positively to patient’s efforts, progress, and achievements	Praises patient’s efforts towards goal efforts irrespective of success	0	0	100
Normalizes and empathizes with goal challenges	Reminds patient that things do not always go to plan and lots of people have setbacks when trying new ways of doing things	4	18	78
Goal adjustment/development	Checks the salience of goals and makes adjustments if required, including adjusting steps towards goals. Re‐establishes commitment using motivational interviewing techniques if necessary	11	0	89
Integration of self‐management approach	Reviews key skills and help patient identify a support network. Discusses maintenance tools and normalizes setbacks	7	78	15
Discussed integration of goals into daily life	Rehearses new skills, such as mindfulness and shifting focus, and explores how these can be extended to other areas of life. Encourages the development of insights and the capacity to self‐initiate change	11	15	74
Uses at least one other metaphor or tool^b^	At any point during the session, at least one other metaphor or tool should be referred to	7	0	93
Session 3				
Responds positively to patient’s efforts, progress, and achievements	Praises patient’s efforts towards goal efforts irrespective of success	0	0	100
Normalizes and empathizes with goal challenges	Reminds patient that things do not always go to plan and lots of people have setbacks when trying new ways of doing things	0	15	85
Integration of self‐management approach	Reviews key skills and help patient identify a support network. Discusses maintenance tools and normalizes setbacks	23	54	23
Discussed integration of goals into daily life	Rehearses new skills, such as mindfulness and shifting focus, and explores how these can be extended to other areas of life. Encourages the development of insights and the capacity to self‐initiate change	23	23	54
Addresses future challenges, including treatment‐seeking	Emphasizes that the patient will face times when they experience pain or other difficulties, and their natural response will be either that treatment did not work or that they need more. Acknowledges that this is normal and reminds them that they have the skills and resources to carry on without further treatment (e.g., PACT patient guide and new skills)	0	46	54
Confident and positive sign off	Positive closure of the therapeutic partnership to help reinforce patient capacity to persist with the tools they have to manage their back pain without needing more health care	0	0	100
Treatment enactment				
Patient refers to using PACT stance/skills – Session 2	At any point during the session, the patient reflects a PACT stance, shows psychological flexibility, shows focus is shifted, used a metaphor/tool between sessions	n/a	n/a	63
Patient refers to using PACT stance/skills – Session 3	At any point during the session, the patient reflects a PACT stance, shows psychological flexibility, shows focus is shifted, used a metaphor/tool between sessions	n/a	n/a	54

^a^ Notice 5 things comprises the following steps: (1) Pause. (2) Look around and notice five objects you can see… (wait at least 10 s). (3) Listen carefully and notice five sounds you can hear… (wait at least 10 s). (4) Notice five things you can feel on the surface of your skin… (wait at least 10 s). (5) And, stop. (6) Consider what happens during this exercise for you.

^b^ At least eight metaphors or tools were taught and/or included in the PACT physiotherapist manual.

^c^ Assessed on a binary scale (yes/no). Remaining items were scored on a 3‐point ordinal scale (‘1 = not completed, 2 = partially completed, and 3 = completed’).

#### ACT competence

An 8‐item scale was developed to assess ACT competence (Table 2). Items were developed based on the ACT for Chronic Pain Adherence Rating Scale (Pincus et al., 2015) and according to guidance for developing ACT fidelity measures (Plumb & Vilardaga, 2010). The scale was simplified to assess the quality of delivery of core processes of ACT delivered by physiotherapists, and content was reviewed by two clinical psychologists specializing in ACT for pain management. Items were assessed on a 5‐point Likert scale: ‘1 = not at all, 2 = a little, 3 = somewhat, 4 = considerably, and 5 = extensively’. Higher scores (maximum score of 40) indicated higher ACT‐specific therapeutic competence. The scale demonstrated good internal reliability in the present study (Cronbach’s alpha 0.84).

**Table 2:** ACT competence scale

	Item	Description
1	Demonstrates a respectful and caring stance	No evidence of judgement or criticism demonstrates an equitable relationship. No arguing, lecturing, coercing, fixing, or convincing
2	Reflects a sense that thoughts and feelings are understandable	People’s thoughts and feelings are determined by their experience, therefore understandable
3	Encourages openness to uncomfortable experiences (such as pain, anxiety, sadness, confusion, fatigue, or others)	The opposite of struggling and being invalidating. Sitting still and pausing when something painful has been said
4	Facilitates patient awareness of thoughts, feelings, or opportunities	Helps people to step back from their thoughts and to reflect on them for what they are, enables the use of mindful techniques
5	Emphasizes a focus on successful attainment of personally meaningful goals as opposed to symptom reduction	Helps people to identify what is important to them and to derive goals that are in line with values, encouragement of seeing values as existing even though they may seem unachievable at this point
6	Deemphasises change in the content of thoughts or feelings as process or outcome	Intrusive thoughts are not facts and do not need a response/to be followed. Worries may remain but function can be improved
7	Organizes or facilitates the active practice of goal‐directed engagement or behaviour change	Helps them to take committed action, e.g., scheduling into their calendar
8	Helps to build behaviour patterns that are integrated across situations and/or persistent	Reconnecting small patterns of behaviour with overarching motivations and purposes to be regularly carried out. Setbacks will happen but now have the ability to manage those

#### Therapeutic alliance

The 5‐item alliance subscale of the adapted Primary Care Therapy Rating Scale (Godfrey, Chalder, Ridsdale, Seed, & Ogden, 2007; Moss‐Morris et al., 2013) assesses therapist and patient contributions to alliance during psychological interventions (Table 3). Items were scored on a 7‐point Likert scale, with anchors at four points: ‘1 = not at all, 3 = somewhat, 5 = considerably, and 7 = extensively’. Higher scores (maximum 35) indicated a stronger therapeutic alliance. The scale demonstrated good internal reliability in the present study (Cronbach’s alpha 0.87).

**Table 3:** Therapeutic alliance scale

	Item	Description
1	Patient self‐discloses thoughts and feelings	Did the patient express their thoughts and feelings to the physiotherapist?
2	Supportive encouragement	Was the therapist supportive of the client by acknowledging the client’s gains during therapy, or by reassuring the client that gains will be forthcoming?
3	Convey understanding	Did the therapist use reflection, paraphrasing, or summarizing to convey that she/he understood the client’s problems?
4	Warmth	Did the therapist convey warmth?
5	Empathy	Was the therapist empathic towards the client (i.e., did she/he convey an intimate understanding of and sensitivity to the client’s experiences and feelings)?

#### Treatment enactment

A single component on the PACT content checklist assessed participant enactment. This was defined as evidence that the participant adopted the PACT stance (e.g., examples of psychological flexibility and shifting focus from pain to activity and goals) and/or skills (using ACT‐consistent metaphors or tools defined in the PACT intervention; Table 1). This component was assessed during sessions 2 and 3 on a binary scale (no and yes). Treatment receipt and enactment (i.e., engagement) were evaluated further using qualitative methods as described below.

### Procedure

#### Quantitative procedures

Two independent assessors, a chartered health psychologist trained to Ph.D. level, and an MSc health psychology student, conducted the fidelity assessment. Assessors were blind to trial outcomes, and physiotherapists and participant identification. They were trained for 2 days on PACT, ACT, and therapeutic alliance principles by a health psychologist (EG), and clinical psychologist (LM). The training was supplemented by a PACT intervention fidelity manual documenting the fidelity framework and methods (Supplementary File S1). This included descriptions and illustrative examples of each assessment item, with definitions of valid or invalid delivery. The fidelity assessment procedure was piloted by the trainers and independent assessors using a randomly selected sample of six audio‐recorded treatment sessions (two each of sessions 1, 2, and 3) that were not included in the primary fidelity assessment sample.

Ratings were conducted independently by each assessor on the full sample of 72 tapes. The PACT intervention content was assessed via a coding framework while listening to an audio‐recorded session, whereas ACT competence and therapeutic alliance were assessed globally after listening to a full session. Overall agreement (Cohen’s Kappa) between raters was 0.85 (95% CI 0.81 to 0.88) for PACT content delivery and 0.30 (95% CI −0.20 to 0.80) for the enactment of treatment skills (Item 7 in sessions 2 and 3) reflecting ‘almost perfect’ and ‘fair’ agreement, respectively (Landis & Koch, 1977). Overall inter‐rater agreement (intraclass correlation coefficients, two‐way mixed effect model, absolute agreement) was 0.47 (95% CI 0.17, 0.67) for the ACT competence scale and 0.39 (95% CI −0.01, 0.63) for the therapeutic alliance scale, reflecting ‘unacceptable’ inter‐rater reliability for both scales (George & Mallery, 2003). To address this, monthly meetings were held between the assessors, which were facilitated by one trainer (EG), to discuss discrepancies and agree on final calibrated scores which were used for analyses.

#### Qualitative procedure

A topic guide was developed by the research team and designed to explore participant experiences of PACT, including treatment acceptability, perceived outcomes, and changes in thoughts and behaviour (Supplementary File S2). Data were collected by an MSc health psychology student who was independent of the PACT study and therefore impartial to and unaware of the trial outcomes. The student was supervised by an academic health psychologist (VW) and the chief investigator (EG), received instruction in qualitative methods and participated in audio‐recorded pilot interviews with feedback from the supervisory team. Following pilot interviews, further refinements to the topic guide were made that simplified content or added prompts to help capture detail or depth of participant accounts. Semi‐structured individual in‐depth interviews guided by the topic guide and lasting up to 60 min were conducted in a private room with participants after they had completed the main endpoint of the trial (after 3 months follow‐up). Interviews were audio‐recorded, then transcribed verbatim.

### Analysis

#### Quantitative analysis of treatment delivery, enactment, therapeutic alliance, and ACT competence

Statistical analyses were conducted using SPSS (version 26) and STATA statistical software (version 12). The Fidelity of PACT content delivery was assessed at two levels: by individual component within sessions and overall, by session. Delivery of each component was rated as not completed, partially completed, and completed for each analysed tape, and proportions were calculated to reflect the frequencies of completed PACT content delivery; a threshold of 80% completed delivery for each component was set as an indicator of high fidelity. Treatment fidelity by session was computed as the proportion of components delivered completely for sessions 1, 2, and 3; a threshold of 80% (of 10, 6, and 6 components, respectively), was set as an indicator of high fidelity and the proportion of sampled sessions achieving high fidelity was reported. Percentage data are also presented for categorical components reflecting treatment enactment. Mean (*SD*) calibrated scores were calculated for continuous data reflecting overall and item‐specific scores on the ACT competence and therapeutic alliance scales.

#### Qualitative analysis of treatment engagement

A secondary analysis of the transcribed data was conducted by two authors (MGH and EG) using a descriptive‐analytical thematic approach (Braun & Clarke, 2006). *A priori* codes were used that reflected receipt or enactment of components on the PACT content checklist. Full transcripts were reviewed and coded deductively (i.e., applying an analyst‐driven approach including the use of *a priori* codes) in chunks (i.e., statement or paragraph units) for evidence of or against participant receipt or enactment (Braun & Clarke, 2012). Coding was conducted by one researcher (MGH) and was validated by a second researcher (EG). The frequencies of coded content were tabulated with illustrative examples used to describe the range and scope of participant engagement. Analysis was conducted using NVivo software version 12 (QSR International Ltd, Southport, UK).

## Results

### PACT intervention content

A total of 72 audio‐recorded PACT treatment sessions (*n* = 32, 27, and 13 samples of sessions 1, 2, and 3, respectively) were assessed for fidelity. The mean number of sessions per physiotherapist was eight (*SD* = 2.73) and mean session lengths were: session 1:59 min, session 2:45 min, and session 3:15 min. The proportions of sampled sessions achieving high treatment fidelity (i.e., 80% of PACT content delivered completely) were: Session 1, 97% (31/32 sessions), Session 2, 81% (22/27 sessions), and Session 3, 77% (10/13 sessions). Of the items assessed, 12 PACT content components were delivered with high fidelity (i.e., delivered completely in at least 80% of sampled sessions), whereas the following five components were not: setting the agenda (session 1), normalizing, and empathizing with goal challenges (session 2), supporting integration of a self‐management approach (sessions 2 and 3), discussing integration of goals into daily life (sessions 2 and 3) and addressing future challenges including treatment‐seeking (session 3; Table 1).

### ACT competence

Individual scale items (Table 2) ranged from mean 1.74 (*SD* 0.73) and 2.40 (*SD* 0.65), indicating therapeutic competence was ‘not at all’ to ‘somewhat’ achieved. The total ACT competence score was mean 16.44 (*SD* = 3.33) corresponding to the scale anchor point: ‘a little’. Scores were consistent between sessions 1 (mean = 17.75, *SD* = 3.33) and 2 (mean = 16.32, *SD* = 2.59), and showed a reducing trend in session 3 (mean = 13.23, *SD* = 2.05).

### Therapeutic alliance

The mean calibrated therapeutic alliance score was 22.6 (*SD* = 4.5), indicating that the scale items were typically rated between the anchor points of ‘somewhat’ and ‘considerably’ (Table 3). The items rated lowest and highest were e*mpathy* (*M* = 3.94, *SD* = 1.33) and s*upportive encouragement* (*M* = 5.08, *SD* = 0.93). Therapeutic alliance scores remained constant across sessions 1 (mean = 22.7, *SD* = 5.2) and 2 (mean = 22.9, *SD* = 3.9), and showed a reducing trend in session 3 (mean = 16.4, *SD* = 3.3).

### Treatment receipt and enactment

Calibrated scores from the PACT intervention content scale demonstrated that treatment enactment (patients reflecting a PACT stance during the session) was observed in 63% and 54% of the sample, during sessions 2 and 3, respectively. In additional qualitative analysis, the frequencies of reports of receipt and enactment of each component with examples are shown in Supplementary File S3. There was evidence of receipt of most (11/17) PACT components. Participants most frequently demonstrated receipt of the PACT patient guide (*n* = 13), identifying SMARTER goals (specific, measurable, action‐orientated, realistic, time‐oriented, emotional, and resonance; *n* = 9) and mindfulness skills (notice five things; *n* = 8). In addition, receipt of exercise guidance emerged as a component received by eight participants; this was not an explicit psychologically informed PACT component but was included as part of the physiotherapy treatment in the PACT patient guide and intervention. All participants except two (G and Q) reported evidence of treatment receipt of at least one component. Participant G recalled discussing exercise and engaging in mindfulness during treatment, but did not demonstrate understanding of these components, whereas participant Q had difficulty recalling PACT content at all; both were sceptical about PACT, did not perceive it as physiotherapy treatment, and had expected manual therapy for their chronic low back pain. Enactment of three PACT components was reported, including the use of the patient guide (*n* = 8), mindfulness (notice five things; *n* = 4), and identifying SMARTER goals (*n* = 3). Enactment of at least one component was demonstrated by nine participants.

## Discussion

This study demonstrated that some, but not all, domains of treatment fidelity were achieved in a pragmatic evaluation of a psychologically informed physiotherapy intervention. Most PACT content (12/17 items) was delivered by physiotherapists with high fidelity in session 1, but five techniques were more problematic and not delivered or subsequently enacted by participants. Participant receipt of delivered treatment content was demonstrated by most (89.5%) participants, and a satisfactory therapeutic alliance was achieved overall. However, ACT competence was low.

PACT content was delivered with high fidelity overall, but this decreased as the intervention progressed, with high fidelity achieved for 97%, 81%, and 77% of the sampled sessions 1, 2, and 3, respectively. Examination of the data at the individual item level revealed that five PACT content components were not adhered to. These components were: the degree to which physiotherapists set the agenda, normalized, and empathized with goal challenges, supported integration of a self‐management approach, discussed the integration of goals into daily life, and addressed future challenges. This content involves the delivery of complex cognitive, emotional, and behavioural skills, with items including multiple sub‐components (e.g., integration of self‐management approach comprises reviewing key skills, identifying a support network, discussing maintenance tools, and normalizing setbacks). Four of these components feature in sessions 2 and/or 3, which were designed to be more flexible to enable tailored responses to individual patient needs. The PACT physiotherapists felt that these sessions were less structured and therefore more difficult to deliver as specified compared with session 1, possibly contributing to the differences in fidelity observed between sessions (Galea Holmes et al., 2020). This may reflect a limitation of core skills training among PACT physiotherapists and a lack of familiarity with incorporating complex psychological skills therapeutically, especially when delivery was remote in session 3. Correspondingly, none of these five components were received or enacted by interviewed participants, which was as expected in the absence of robust and consistent delivery.

Physiotherapists used a content checklist to facilitate and standardize treatment delivery, which may have contributed to the high fidelity observed for some components (Bellg et al., 2004; Borrelli et al., 2005). However, the use of treatment checklists and manuals in behavioural interventions may overlook unique provider contributions, compromise the authenticity of provider–patient interactions, and inhibit patient engagement, and are not widely used by psychologists (Addis & Krasnow, 2000; Becker, Smith, & Jensen‐Doss, 2013). By contrast, Kendall et al. suggest that, when adopted flexibly to guide interventions, checklists can support evidence‐based, patient‐centred, and individualized treatment (Kendall & Beidas, 2007; Kendall, Gosch, Furr, & Sood, 2008). Consistently, PACT physiotherapists felt reassured using the checklist, and with experience desired greater flexibility and autonomy in their treatment delivery (Galea Holmes et al., 2020). Therefore, a simplified checklist that supports adherence to the essential intervention content and reinforces learning, while balancing flexibility is recommended when implementing psychologically informed physiotherapy, such as PACT, outside of a randomized controlled trial. It may be feasible to specify essential and optional components, depending on the needs of individual participants, to facilitate this.

Participant engagement with PACT varied across intervention content and between individuals, with mixed results for treatment receipt and poor evidence of enactment. Treatment receipt and enactment by participants rely on robust treatment delivery; it is not surprising therefore that, based on participant accounts, the PACT patient guide, mindfulness skills (in particular, notice five things), and SMARTER goal setting were received by most individuals and enacted by some. These are examples of discrete and structured intervention components, which were also delivered consistently by physiotherapists, and may have been easier for participants to recount, understand, and adopt. For example, the mindfulness skill, ‘notice five things’, was taught to PACT physiotherapists through experiential learning, and comprised a sequence of instructions with examples to perform and deliver the skill. This might have enhanced delivery fidelity and participant engagement. Similarly, SMARTER goal setting builds on existing practice and was conducted interactively using the patient guide during session one, integrating these intervention components and supporting physiotherapist and participant interaction. However, other PACT content was received by ≤3 participants, and evidence of enactment was even lower, suggesting that the intervention was, overall, not well understood or adopted into daily life by participants. Strategies to increase receipt and subsequent enactment of a wider scope of PACT content could be incorporated, such as using role‐play with participants to coach and feedback on skills, collecting and reviewing self‐monitoring data, and assessing participants knowledge and confidence to perform skills (Borrelli, 2011); however, these strategies would require increased time and resource that may be beyond the scope of a brief intervention.

The findings discussed above suggest greater challenges in delivering and evaluating the most complex, nuanced, or abstract PACT content. This is consistent with our finding that overall, physiotherapists achieved only ‘a little’ ACT competence. This finding suggests challenges in supporting physiotherapists to deliver a complex ACT‐based psychological approach following a brief training programme. In addition, this finding contrasts with PACT physiotherapists own reports of perceived self‐confidence and ‐competence in their skills (Galea Holmes et al., 2020). The PACT intervention was developed for delivery by non‐psychologists and incorporated techniques that extended physiotherapists' core skills. Developing high‐level proficiencies may require more in‐depth, ongoing learning and supervision (Holopainen et al., 2020); moreover, consistent feedback and clear benchmarks might be helpful to align perceived and observed competence. However, more intensive PACT training may not be feasible in the context of time‐ and resource‐constrained service delivery settings and novel treatments need to be designed to be implementable into routine clinical practice (O'Cathain et al., 2019).

Competence was particularly low during the remotely delivered session 3, which PACT physiotherapists found less structured and more challenging than face‐to‐face sessions (Galea Holmes et al., 2020). It is possible that because of changes to practice initiated in response to the COVID‐19 pandemic, clinicians may be more familiar with remote delivery, so this becomes less of an issue. Alternatively, it may be that more specific training or adaptations to the content of this session are needed to facilitate fidelity, or more online resources, such as pre‐recorded videos to demonstrate a session from start to finish, might be helpful. In addition, strategies to facilitate completely remote delivery of PACT should be established and evaluated for wider roll‐out in the post‐COVID era.

Therapeutic alliance scores were above the scale midpoint for sessions 1 and 2, suggesting it was observed at least ‘somewhat’ to ‘considerably’, but decreased during session 3. Patient‐centred interaction styles, including empathy, attentiveness, and active listening are associated with the therapeutic alliance, including how it is conceptualized in physiotherapy practice (Pinto et al., 2012; Søndenå, Dalusio‐King, & Hebron, 2020). Empathy was the lowest‐scoring component on the therapeutic alliance scale and is a quality that underpins the ACT approach, suggesting a target for in‐depth training and development. The PACT intervention provided general and ACT‐specific communication skills that may have promoted therapeutic alliance, including the use of metaphors, normalizing, and empathizing with experiences, and collaborative discussions about individual values and goals which featured in the first two treatment sessions; however, these skills may require more specific attention to promote the therapeutic alliance. Empathy and other aspects of the therapeutic alliance were particularly challenging to achieve during session 3, which was brief (i.e., 20 min) and delivered remotely by telephone. This session lacked explicit characteristics of the intervention designed to support therapeutic alliance including sessions of up to 60 min in a private room. Privacy and time are factors that provide a safe space for patients to share emotional concerns confidentially with their physiotherapist and an appropriate context where the therapeutic alliance can be fostered (Moore et al., 2020; Søndenå et al., 2020). Alternative approaches to achieving therapeutic alliance during remote delivery, for example, using video‐conferencing or nuanced communication techniques (Lozano et al., 2015), could further improve PACT treatment.

Our findings suggest a need to address challenging intervention content that draws on intricate interpersonal, emotional, and behavioural techniques to achieve treatment fidelity. In particular, ensuring physiotherapists and other non‐psychologist healthcare professionals demonstrate competence in core skills is crucial to ensuring safe and effective psychologically informed practice. With adequate training and resources, psychological approaches can be delivered safely and effectively by physiotherapists (Hall et al., 2018). Consistently, the PACT trial found no adverse events attributable to treatment, consistent with other trials evaluating psychologically informed physiotherapy, and future evaluations should continue to evaluate and report any potential risks and harms (Zhang, Jiang, Young, & Li, 2019).

Strengths of this study include the use of qualitative and quantitative methods to evaluate treatment fidelity, and a comprehensive assessment of physiotherapist delivery and patient engagement. The methods for developing and conducting the PACT fidelity evaluation align with recently published recommendations by Walton et al. (2020), which include reviewing previous measures, developing a framework of intervention content, developing fidelity checklists, and coding guidelines, obtaining feedback on checklists and guidelines, and piloting and refining materials and procedures. We employed a systematic approach that drew on existing measures and a tailored checklist and incorporated qualitative evidence. In addition, comprehensive training, and guidelines, documented in a manual, were provided to independent raters, who piloted materials and procedures with the training team. However, rater agreement on scales measuring therapeutic alliance, ACT competence and patient enactment was low, and required consensus meetings to achieve final calibrated scores, which is a confounding factor that may have influenced our results. This reflects well‐documented challenges in assessing treatment fidelity, including therapeutic competence (Fairburn, 2011), but could be addressed by resource‐intensive strategies that were outside the scope of this study including additional rater training, frequent meetings to align performance and benchmarks, including employing more than two raters, and prior research to establish validity and reliability of assessment tools (e.g., Feely, Seay, Lanier, Auslander, & Kohl, 2018; Kramer Schmidt, Andersen, Søgaard Nielsen, & Moyers, 2019).

A limitation of this study was that the evaluation was observer‐led, and other assessments including quantitative self‐report measures of participant and provider experiences are recommended which could have enabled a more robust triangulation of findings. The imbalance in sampled sessions may have introduced some bias in the overall ratings, although this is small and unlikely to impact the main conclusions; in particular, the smaller sample of session 3 tapes contributed to increased uncertainty in the estimates for this session. We conducted a secondary analysis of qualitative data that was not intended solely to evaluate patient engagement; therefore, the topic guide did not include questions on receipt or enactment explicitly and included prompts for some but not all treatment content which may have contributed to a bias in participant accounts. We adapted established measures of therapeutic alliance and ACT competence using the best available tools at the time; however, it was not feasible to refine the scales through rigorous development and psychometric evaluation. Overall, the study was limited by common challenges including a resource‐intensive process with no additional funding and the need to develop or adopt measures that were fit for purpose. Attention needs to be paid to funding trial process evaluations appropriately and simplifying methods and treatments where possible to enable evaluation of fidelity and successful implementation of treatment.

### Conclusions

A comprehensive, mixed‐methods fidelity evaluation of a psychologically informed physiotherapy treatment for individuals with chronic low back pain demonstrated some challenges in treatment delivery and engagement. Overall, PACT treatment sessions were delivered with high fidelity, but not all PACT components were delivered with fidelity and received by participants. Some domains of treatment fidelity were achieved but others were more problematic, and evidence of participant enactment was low. The therapeutic alliance was observed but results suggest a need to improve empathy during the therapist–patient interaction, and the physiotherapist’s ACT‐specific therapeutic competence was low. Physiotherapists delivering PACT may benefit from a simplified intervention including core and optional components, enhanced skills training, and more frequent opportunities to apply psychological approaches to hone expertise when working therapeutically. Additional structure and ongoing support may be required to implement more complex components and thus improve competence and engagement.

## Conflicts of interest

All authors declare no conflict of interest.

## Author contribution

**Melissa Galea Holmes:** Formal analysis (equal); Writing – original draft (equal); Writing – review & editing (equal). **Vari Wileman:** Conceptualization (equal); Formal analysis (equal); Methodology (equal); Project administration (equal); Supervision (equal); Writing – original draft (equal); Writing – review & editing (equal). **Shaira Hassan:** Investigation (equal); Project administration (equal); Writing – original draft (equal); Writing – review & editing (equal). **Julie Denning:** Investigation (equal); Writing – review & editing (equal). **Duncan Critchley:** Conceptualization (equal); Funding acquisition (equal); Methodology (equal); Supervision (equal); Writing – review & editing (equal). **Sam Norton:** Formal analysis (equal); Funding acquisition (equal); Supervision (equal); Writing – review & editing (equal). **Lance McCracken:** Funding acquisition (equal); Methodology (equal); Supervision (equal); Writing – review & editing (equal). **Emma Godfrey:** Conceptualization (equal); Funding acquisition (equal); Investigation (equal); Methodology (equal); Supervision (equal); Writing – review & editing (equal).

## Supporting information

Supinfo S1 PACT Treatment Fidelity Measure.

Supinfo S2 Patient interview topic guide.

Supinfo S3 Participant engagement: receipt and enactment of PACT components.

## Data Availability

The data that support the findings of this study are available from the corresponding author upon request subject to appropriate data sharing permissions.
