# Supplementary material for: Physiotherapy informed by Acceptance and Commitment Therapy for chronic low back pain: A mixed‐methods treatment fidelity evaluation
Source: Br J Health Psychol. 2022 Feb 3;27(3):935–55. doi: 10.1111/bjhp.12583 (PMC9540449; doi:10.1111/bjhp.12583)
Supplement: Supplementary file 1 — Supinfo S1 PACT Treatment Fidelity Measure. [file BJHP-27-935-s002.doc]

| PACT© |
| --- |
| **PACT Treatment Fidelity Measures** |

# PACT Treatment Fidelity Measure

### Guidance for Treatment Fidelity Raters

1. Raters should listen to the tapes and take notes while listening. The ACT Therapeutic Scale and Therapeutic Alliance Scale should both be completed at the end.
2. Raters should be careful and conscientious when listening to the tapes - rating requires good judgement and should not be influenced by first impressions.
3. Raters should assess the *extensiveness* (frequency and intensity = extensiveness) of the behaviour rather than the quality, although it is acknowledged that the two are not totally distinct.
4. Raters should rate what they actually hear and not what they think ought to have occurred.
5. If something does not occur, it should be rated as 1. In order to rate an item greater than 1 – the rater must hear a specific example of the behaviour being rated.
6. Raters should choose the more extreme score when they hesitate between 2 ratings to avoid regression to the mean. They should also be aware of their own tendency to be lenient, extreme or rate in the middle.
7. The rater should be aware of the concept of a good alliance and rate according to this, rather than the patient’s *ability* to form a good alliance.
8. The rater must apply the same standards for rating an item regardless of:
9. What ratings were given to other items or in other sessions
10. How much the rater likes the therapist
11. Other behaviour the therapist engaged in during the session
12. Whether the rater believes the behaviour being rated was a good or bad thing to do
13. How skilled the rater believes the therapist to be.

# The PACT study

## PACT Treatment Fidelity Measure

**Assessor ID:**

**Audio filename:**

**Date:**

**Please tick appropriate PACT Session (1, 2 or 3). 4 and 5 are compulsory for all assessments.**

1. PACT Session 1 (p.6)

2. PACT Session 2 (p.10)

3. PACT Booster call (p.12)

4. ACT Fidelity Scale (p.14)

5. Therapeutic Alliance Scale (p.16)

## PACT Session 1

**Length of treatment session** Hrs Mins

**Not completed** **1**

**Partially completed** **2**

| **PACT treatment task** | **1** | **2** | **3** |
| --- | --- | --- | --- |
| **1. Sets the agenda:** outlines structure, schedule and delivery of treatment. |  |  |  |
| **2. Conducts brief physical assessment** |  |  |  |
| **3. Covers feedback:** explains that no serious medical problems have been uncovered and that it is safe to resume activities. |  |  |  |
| **4. Shifts focus from pain to function:** rather than struggling with pain, suggests openness to another approach and presents the goal of PACT, to help people function better especially in the areas that are important to them. |  |  |  |
| **5.** a) **Helps patient identify SMARTER goals:** engages patient in identifying core values and setting related goals. |  |  |  |
| b) Breaks goals down into small steps. |  |  |  |
| c) Records agreed goals in the patient manual. |  |  |  |

**Completed** **3**

**1.** Physiotherapist explains what will happen in the session. Provides a warm and enthusiastic introduction and welcome.

**5.** a) Physiotherapist emphasises the importance of goals that matter just to the patient, and related activities that they have missed due to their back pain. Invites patient to think about what matters most to them and what activities that they would like to return to doing. Uses the Patient Specific Functional Scale (PSFS) items to help patient identify value based goals if required.

**2.** Patient has the experience of being clinically assessed. Physiotherapist communicates with patient to identify and rule out red flags.

**3.** Physiotherapist reassuringly confirms that the thorough clinical investigation found no serious problems requiring medical attention. Physiotherapist supportively explains that increasing level of activity will not harm and instead that pursuing a gradual return to activity is the healthiest thing to do.

**4.** Physiotherapist offers patient an alternative approach, one that doesn’t involve finding out what’s happening with, or attempts to fix, their back pain. Instead of focussing on making the pain go away, simply to carry on functioning instead, which they are safe to do. Physiotherapist might also help patient reflect on previous attempts to reduce pain that might not have been successful.

b) Physiotherapist encourages patient to identify manageable and achievable goals. Promotes a slow and steady approach to avoid a boom or bust scenario.

c) Physiotherapist asks the patient to write down their goals in their PACT Patient Guide and reminds the patient to refer to this at home.

## PACT Session 1 contd.

**Not completed 1**

**Partially completed 2**

**Completed 3**

| **PACT treatment task** | **1** | **2** | **3** |
| --- | --- | --- | --- |
| **6.**  **Addresses barriers to goal attainment:**  Physiotherapist encourages patient to consider and prepare for potential barriers to goal fulfilment. Implements strategies to promote openness, awareness and engagement, for example mindfulness exercises and action plans in response to potential barriers. |  |  |  |
| **7. Taught Notice 5 Things** 1. Pause. 2. Look around and notice five objects you can see… (wait at least 10 seconds). 3. Listen carefully and notice five sounds you can hear… (wait at least 10 seconds). 4. Notice five things you can feel on the surface of your skin… (wait at least 10 seconds). 5. And, stop. 6. What happens during this exercise for you? |  |  |  |
| **8. Uses at least one other metaphor or tool** (e.g. Focus, Get off your buts, Quicksand, Fire Alarm, Sleep, Hamster on a wheel, Swamp, Passing train).  Metaphor or tool used  ………………………………………...................................................................... |  |  |  |
| **9. Provides PACT Patient Guide** | **Yes 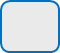 No 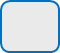** | | |
| **10. Handshake agreement:** making a public commitment to goals. | **Yes 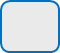 No 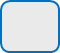** | | |

**6.** Physiotherapist explains that it is normal to get caught up in our thoughts and lose track of what is around us. Facilitates patient to create action plans and explains useful exercises to use when the patient is struggling, feeling stressed and anxious, which helps bring them back to the here and now, and refocus on what is important to them.

**7.** Physiotherapist demonstrates Notice 5 Things and reinforces how the patient can use this skill anytime on their own to help when they are struggling with their pain.

**8.** At any point during the session, the physiotherapist should refer to one other metaphor or tool. Note which metaphor or skill is used.

**10.** Tick Yes if the physiotherapist and patient shake hands or make a verbal agreement on the agreed goals.

**9.** Tick Yes if the physiotherapist provides the patient with the PACT Patient Guide

## PACT Session 2

**Length of treatment session** Hrs Mins

**Not completed 1**

**Partially completed 2**

**C**ompleted 3

| **PACT Treatment task** | **1** | **2** | **3** |
| --- | --- | --- | --- |
| **1. Responds positively to patient’s efforts, progress and achievements:** praises patient’s efforts towards goal efforts irrespective of success. |  |  |  |
| **2. Normalises and empathises with goal challenges:** reminds patient that things do not always go to plan and lots of people have setbacks when trying new ways of doing things. |  |  |  |
| **3. Goal adjustment/development:** checks the salience of goals and makes adjustments if required, including adjusting steps towards goals.  Re-establishes commitment using motivational interviewing techniques if necessary. |  |  |  |
| **4. Integration of self-management approach:**  reviews key skills and helps patient identify a support network. Discusses maintenance tools and again normalises setbacks. |  |  |  |
| **5. Discussed integration of goals into daily life:** discusses the integration of new activity patterns into daily life. Rehearse new skills, such as mindfulness and shifting focus and explores how these can be extended to other areas of life. Encourages the development of insights and the capacity to self-initiate change. |  |  |  |
| **6. Uses at least one other metaphor or tool:** (e.g. Focus, Get off your buts, Quicksand, Fire Alarm, Sleep, Hamster on a wheel, Swamp, Passing train).  Metaphor or tool used  ………………………………………...................................................................... |  |  |  |

**1.** Physiotherapist facilitates an encouraging and positive conversation about efforts made and progress towards goals. Highlights the achievements and benefits as well as acknowledging setbacks. If patient has not managed to even attempt goals, physiotherapist still maintains a positive stance, rewarding them for attending the second session and encouraging towards future efforts.

**5.** To further prepare patient for self-management, talks with them about their daily life (work, family, social life) and how their chosen goals fit more broadly? What can they do to make it easier? Boosts the patient’s confidence that they have the skills and capacity to self-initiate change

**2.** Physiotherapist reassures patient that it is normal to find goals challenging and have setbacks and encourages them to keep trying. Reminds the patient that everyone experiences challenges when trying something new. It’s normal to experience disappointment with setbacks but they can try again.

**4.** Physiotherapist helps them to think about the people around them who can support their pursuit of goals. Helps the patient identify skills that will help them remain focussed on achieving their goals.

**6.** At any point during the session, the physiotherapist should refer to another metaphor or tool. Note which metaphor or skill is used.

**3.** Asks the patient about how important the goal still is to them, established if a goal might need adjusting or a new goal agreed? The goal itself might remain unchanged but the steps towards the goal might need adjusting. Keeps moving in small steps toward goals, troubleshooting or preventing the effects of barriers. Recognises and appreciates patient uncertainty and motivates them to continue their commitment towards their value-based goals. Rolls with resistance, gently but steadily encourages commitment, openly recognising potential setbacks that might present.

## PACT Booster session

**Length of treatment session
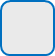
** Hrs **
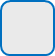
** Mins

**Not completed 1**

**Partially completed 2**

**Completed 3**

| **PACT Treatment task** | **1** | **2** | **3** |
| --- | --- | --- | --- |
| **1. Responds positively to patient’s efforts, progress and achievements:** praises patient’s efforts towards goal efforts irrespective of success. |  |  |  |
| **2. Normalises and empathises with goal challenges:** reminds patient that things do not always go to plan and lots of people have setbacks when trying new ways of doing things. |  |  |  |
| **3. Integration of self-management approach:**  reviews key skills and helps patient identify a support network. Discusses maintenance tools and again normalises setbacks. |  |  |  |
| **4. Discussed integration of goals into daily life:** discusses the integration of new activity patterns into daily life. Rehearse new skills, such as mindfulness and shifting focus and explores how these can be extended to other areas of life. Encourages the development of insights and the capacity to self-initiate change. |  |  |  |
| **5. Addresses future challenges, including treatment seeking:**  emphasises that the patient will face times when they experience pain or other difficulties, and their natural response will be either that treatment did not work or that they need more. Acknowledges that this is normal and reminds them that they have the skills and resources to carry on without further treatment (e.g. PACT Patient Guide and new skills). |  |  |  |
| **6. Confident and positive sign off:**  positive closure of the therapeutic partnership to help reinforce their capacity to persist with the tools they have to manage their back pain without needing more health care. |  |  |  |

**1.** Physiotherapist facilitates an encouraging and positive conversation about efforts made and progress towards goals. Highlights the achievements and benefits as well as acknowledging setbacks. If patient has still not managed to attempt goals, physiotherapist maintains a positive stance, rewarding them for attending both PACT sessions so far and encouraging towards future efforts.

**2.** Physiotherapist reassures patient that it is normal to find goals challenging and have setbacks and encourages them to keep trying. Reminds the patient that everyone experiences challenges when trying something new. It’s normal to experience disappointment with setbacks but they can try again.

**5.** Reminds the patient that they will certainly experience hurdles and setbacks, this is normal for everyone. Encourages them to prepare for this with action planning. Praises them for the efforts and successes so far and commends their grasp of the new skills learnt. Makes sure they know that they are skilled enough to continue this journey on their own now. Understands that they might have some doubts, this is normal. Tells the patient that they can still think about their doubts but carry on regardless. Does not argue against doubts, simply reflects that doubts are normal and that doubts and success can both happen at the same time.

**6.** Physiotherapist leads an ‘all positive’ ending to their partnership. Does not reflect on lack of time or desire to meet/talk again. The treatment has come to its intended close and the patient is fully skilled and able to self-manage.

**3.** Physiotherapist helps them to think about the people around them who can support their pursuit of goals. Helps the patient identify skills that will help them remain focussed on achieving their goals.

**4.** To further prepare patient for self-management, talks with them about their daily life (work, family, social life) and how their chosen goals fit more broadly? What can they do to make it easier? Boosts the patient’s confidence that they have the skills and capacity to self-initiate change.

## ACT Fidelity Scale

| 1 | 2 | 3 | 4 | 5 |
| --- | --- | --- | --- | --- |
| Not at all | A little | Somewhat | Considerably | Extensively |

|  | **1** | **2** | **3** | **4** | **5** |
| --- | --- | --- | --- | --- | --- |
| 1. Demonstrates a respectful and caring stance |  |  |  |  |  |
| 1. Reflects a sense that thoughts and feelings are understandable |  |  |  |  |  |
| 1. Encourages openness to uncomfortable experiences (such as pain, anxiety, sadness, confusion, fatigue or others) |  |  |  |  |  |
| 1. Facilitates patient awareness of thoughts, feelings or opportunities |  |  |  |  |  |
| 1. Emphasises a focus on successful attainment of personally meaningful goals as opposed to symptom reduction |  |  |  |  |  |
| 1. Deemphasises change in content of thoughts or feelings as process or outcome |  |  |  |  |  |
| 1. Organises or facilitates active practice of goal-directed engagement or behaviour change |  |  |  |  |  |
| 1. Helps to build behaviour patterns that are integrated across situations and/or persistent |  |  |  |  |  |

##

1. Respectful of the client’s ability to make their own change, and manage their own circumstances. ‘I respect your capacities to make a success of this’. Pushing too hard is disrespectful. It is stepping back from notions such as ‘I can fix this for you’. Reflected in language and tone of voice, openness to patient’s point of view and respects their perceptions, does not disregard emotions or thoughts. No evidence of judgement or criticism demonstrates an equitable relationship: no arguing, lecturing, coercing, fixing or convincing.

7. Helps them to take committed action. Facilitates taking action in and out of the session’ – identifying a goal or a value, putting something in a calendar , let’s do a stretching exercise right now, Notice 5 Things (or another skill) are all actions. Patient taking action to write not the physio writing it for them, but literacy should be taken into account. Patient read back the goals. ‘Walking seems to be an interest of yours, I wonder how we could put that into action’ – it should not be the physio stating or doing all of the defining.

6. Intrusive thoughts, ‘my pain is terrible’, don’t try to change thought, just accept that they have that thought and try to get a different relationship/reaction to that thought, as thoughts can reoccur – what other thoughts can there be? ‘Notice they are just thoughts, they’re not facts, you don’t have to respond to them’. Makes a difference between wrestling and taking steps in your behaviour.e.g. uses metaphors such as the bus to enable noticing rather than changing, links the thoughts that people have with how they might typically respond, asks if its workable and queries if responding has helped: Emphasises change in response to thoughts rather than altering thoughts, enables choice to follow or not follow thoughts, models how to acknowledge thoughts and feelings without fixing them, by exploring without problem solving. ‘I may not have talked you out of worries but we can still move forward’ ‘your worries may still be there but we can help improve your functioning’

8. It’s all about you, there may be setbacks but you’ve gained a better ability to manage those, you won’t necessarily need to seek further help’. ‘Can you do Notice 5 things here and at home’, ‘difficulties are going to happen’ (persistent), ‘you’ve done this once a day as practice, how can we make this part of your life regularly’, once a skill is acquired applying that skill where needed, helping someone to identify relevant situations to use it further, anything motivating (persistence creating), reconnecting small patterns of behaviour with overarching motivations and purposes, is it important to the person.e.g. encourages the use of tools and techniques to extend beyond singular goals in order to pursue values.

2. People’s thoughts and feelings are determined by their experience, this is the sense in which they are understandable. ‘You are not to blame for your thoughts and feelings, your thoughts and feelings just happen given your history, they’re entirely understandable with respect to the experiences you’ve had’. Understandable is given a learning history point of view, in a non-blaming way, helps people to not make a big deal/feel guilty about their thoughts and feelings as they are legitimate and understandable given their experiences. Acknowledges emotions ‘I can see it is distressing/painful for you’, use of metaphors to demonstrate understanding, e.g. hamster on a wheel, sleep metaphor, normalises experience.

3. Encourages openness to uncomfortable experiences, the opposite of struggling. Pointing to struggling not working very well from someone’s experience would encourage openness. Look at it functionally rather than assessing if the patient was told, as PACT treatment time is short for the patient to come to the realisation themselves – is what was done and the quality of what was done likely to encourage openness? Or is it shutting down and being invalidating? What was said and how it was said. Normal openness and encouraging things are sitting still and pausing for a couple of seconds, when something painful has just been said or expressed (normal and encouraging openness), without saying anything (silence).

4. Helps people to step back from their thoughts and to reflect on them for what they are, enables the use of mindful techniques such as Notice 5 things to increase awareness, highlights where there might be two opposing thoughts existing at the same time, asks patients to think about things from someone else’s perspective in order to separate self from thoughts. ‘what would you advise your family or friends to do?’ ‘why do you advise yourself differently? Uses ‘get off butts’ to enable flexibility and enable opportunity. Uses more direct and less abstract questions e.g. rather than, ‘if you were to go shopping’, says instead ‘Imagine you were going to go shopping right now, what feeling/thought shows up? What would it mean to you, what could you gain, what could you achieve by going shopping?’

5. Helps set the agenda. ‘What do we focus on? Is this about fixing pain or achieving goals? If we really want to succeed, goals should be focused on’. Helps people to identify what is important to them and to derive goals that are in line with values, encouragement of seeing values as existing even though they may seem unachievable at this point. ‘If you got rid of your pain, what would you be doing?’ –focus on functioning and pursuit of values.

## Therapeutic Alliance Scale (TASC-6*)

| 1. Patient self-discloses thoughts and feelings:   Did the patient express their thoughts and feelings to the physiotherapist? | | | | | | |
| --- | --- | --- | --- | --- | --- | --- |
| 1 | 2 | 3 | 4 | 5 | 6 | 7 |
| Not at all |  | Some |  | Considerably |  | Extensively |

| 1. Supportive encouragement:   Was the therapist supportive of the client by acknowledging the client’s gains during therapy, or by reassuring the client that gains will be forthcoming? | | | | | | |
| --- | --- | --- | --- | --- | --- | --- |
| 1 | 2 | 3 | 4 | 5 | 6 | 7 |
| Not at all |  | Some |  | Considerably |  | Extensively |

| 1. Convey understanding:   Did the therapist use reflection, paraphrasing or summarizing to convey that she/he understood the client’s problems? | | | | | | |
| --- | --- | --- | --- | --- | --- | --- |
| 1 | 2 | 3 | 4 | 5 | 6 | 7 |
| Not at all |  | Some |  | Considerably |  | Extensively |

| 1. Warmth:   Did the therapist convey warmth? | | | | | | |
| --- | --- | --- | --- | --- | --- | --- |
| 1 | 2 | 3 | 4 | 5 | 6 | 7 |
| Not at all |  | Some |  | Considerably |  | Extensively |

| 1. Empathy:   Was the therapist empathic towards the client (i.e. did she/he convey an intimate understanding of and sensitivity to the client’s experiences and feelings)? | | | | | | |
| --- | --- | --- | --- | --- | --- | --- |
| 1 | 2 | 3 | 4 | 5 | 6 | 7 |
| Not at all |  | Some |  | Considerably |  | Extensively |

| 6. Patient and therapist agree on the kind of changes to make:  To what extent did the patient and therapist reach agreement on the best way forward? | | | | | | |
| --- | --- | --- | --- | --- | --- | --- |
| 1 | 2 | 3 | 4 | 5 | 6 | 7 |
| Not at all |  | Some |  | Considerably |  | Extensively |

***Adapted from [1, 2]**

**1.** Patient feels comfortable enough to express what they think and feel about their back pain and what it prevents them from doing.

**2.** Measures how supportive therapist is of client’s efforts i.e. Physiotherapist points out & acknowledges positive changes, encourages client to continue as more will come.

**4.** This is equated with “unconditional positive regard” as defined by Rogers. i.e. deep and genuine caring about the client as a person, without any judgement. Physiotherapist communicates with natural warmth and compassion, caring that the patient is a person not just a client, does not judge in any way.

**5.** To what extent did the physiotherapist convey to patient that they understand what the client is experiencing and feeling and how much it means to them. (This item reflects the emotional level in which the physiotherapist interprets and responds to what the patient has said, in contrast to *item 3. Understanding*)

There are subtle differences between all these items. Broadly, the therapeutic alliance is generally understood to be made up of three key aspects: patient commitment and working capacity, working strategy consensus, and therapist understanding and involvement. The items below are about the working strategy consensus i.e. for any therapy to be successful, the therapist and client need to agree on the strategy to employ and the goals they’re trying to achieve in therapy. To rate it, we need to look at what the patient’s needs or wants to get out of therapy and what the therapist is providing and the concordance or discrepancy between them. Client and therapist agree on the kind of changes to make – i.e. share same understanding about how people get help with this problem and therefore what changes to make. Client and therapist share same sense of how to proceed  - i.e. client and therapist degree of agreement, implicitly or explicitly, about how to move forward Client and therapist agree on salient themes  - i.e. agree what’s important and work together for this, not at cross purposes

**3.** Physiotherapist demonstrates to the patient that they understand their problems and feelings. Achieves this though reflection, paraphrasing or summarising what they patient tells them. (This item reflects the accuracy in which the physiotherapist interprets what the patient has said, in contrast to *item 5.Empathy*)

**6.** To what extent physiotherapist and patient agree on the kind of changes to be made and how best to proceed. The discussion should be balanced between both parties and not at cross purposes.

## Notes

## References

1. Godfrey, E., et al., *Investigating the active ingredients of cognitive behaviour therapy and counselling for patients with chronic fatigue in primary care: developing a new process measure to assess treatment fidelity and predict outcome.* British Journal of Clinical Psychology, 2007. **46**(3): p. 253-272.

2. Moss-Morris, R., et al., *A randomized controlled trial of cognitive behavioral therapy (CBT) for adjusting to multiple sclerosis (the saMS trial): does CBT work and for whom does it work?* Journal of consulting and clinical psychology, 2013. **81**(2): p. 251.

##

## 
