# Supplementary material for: Physiotherapy informed by Acceptance and Commitment Therapy for chronic low back pain: A mixed‐methods treatment fidelity evaluation
Source: Br J Health Psychol. 2022 Feb 3;27(3):935–55. doi: 10.1111/bjhp.12583 (PMC9540449; doi:10.1111/bjhp.12583)
Supplement: Supplementary file 2 — Supinfo S2 Patient interview topic guide. [file BJHP-27-935-s003.doc]

Thank you for agreeing to take part in this interview today, which focus on your experiences of taking part in the PACT study. This study is testing a new treatment for chronic back pain and consists of two 1hour treatment sessions with a physiotherapist in a private room, followed by one treatment session a month later via follow up telephone call.

This interview will last about an hour. I will be taking notes during the interview and I would also like to record the interview so that I do not miss anything you say if that is OK. Everything that you tell me will be kept completely confidential and you w.ill not be identified in any way as we won’t use anyone’s name when we write up the results

*I’d like to start off by asking you about your general impression (experiences) of the PACT study*

What motivated or encouraged you to take part in the PACT treatment study?

What were you expecting from the treatment? (prompt: to what extent would you say your expectations were met?)

What was it like taking part in the PACT study?

In what ways did the treatment benefit you during or after the sessions? (prompt: any benefits for you emotionally? Physically? In your daily life and activities…?)

How did the PACT treatment compare to any previous treatments you may have had for back pain?

How has the way you respond to back pain changed since having this treatment?

Acceptability of specific elements of treatment (treatment components and context)

*I would like to now talk to you about the treatment you received in some more detail.*

What aspects of the treatment did you find particularly helpful?

What aspects of the treatment did you find particularly unhelpful?

How did you feel about having the treatment in a private room?

How did you feel about the two treatment sessions being one hour long? (prompt: longer than normal physio sessions, was this acceptable to you, and why?)

How did you feel about having these two treatment sessions a few weeks apart? (prompt: to what extent was this acceptable to you, and why?)

Thinking about the first treatment session, how did you find this? (probe: what benefits did you get from the first treatment session? To what extent were you satisfied with the first treatment session, and why?)

Prompts: To what extent was the discussion about accepting more and struggling less with pain helpful? How was learning new skills like mindfulness helpful? How was focusing on what was really important to you helpful? How was setting goals helpful?

Thinking about the second treatment session, how did you find this? (probe: what benefits did you get from the second treatment session? To what extent were you satisfied with the second treatment session, and why?) Prompts: To what extent was working towards being more engaged and active helpful? How was working on your goals and addressing barriers to achieving them helpful?...was reviewing your goals helpful?

How did you find the follow-up telephone call you received? Briefly tell me about the call you received?

(prompts: To what extent were you satisfied with this type of treatment? How did it compare to seeing your physio face to face?)

What are your views on the patient guide/booklet you received? Prompt: Show copy. Did you read/use it? Are you still using it at all? Would you have liked to have this in another format e.g. on-line website or app etc

Would you recommend this treatment to other people with back pain? (prompt: please could you explain to me your reasons).

How could we improve this treatment to better meet the needs of people with back pain?

Self-management (How have participants changed their behaviour/activity?)

To what extent did the PACT treatment give you any new tips about how to manage your back pain yourself?

How helpful did you find this advice?

How have you incorporated any of it into your everyday life?

Are you currently seeking any further treatment for your back pain? (prompt: please could you explain to me your reasons).

Overall, how satisfied are you with the treatment you have received?

Is there anything else you would like to add?

Thank you very much for taking part in this interview
