# Supplementary material for: Physiotherapy informed by Acceptance and Commitment Therapy for chronic low back pain: A mixed‐methods treatment fidelity evaluation
Source: Br J Health Psychol. 2022 Feb 3;27(3):935–55. doi: 10.1111/bjhp.12583 (PMC9540449; doi:10.1111/bjhp.12583)
Supplement: Supplementary file 3 — Supinfo S3 Participant engagement: receipt and enactment of PACT components. [file BJHP-27-935-s001.docx]

| **Supplementary file 3** Participant engagement: receipt and enactment of PACT components | | | |
| --- | --- | --- | --- |
| PACT treatment task | Description | Participants reporting receipt, n | Participants reporting enactment, n |
| 1. Exercises |  | 8 | 11 |
| 1. Sets the agenda | Outlines structure, schedule and delivery of treatment; establishes foundation for good therapeutic alliance |  |  |
| 1. Conducts brief physical assessment | Identify and rule out red flags | 3  “She put me at ease in terms of reassuring me that that there wasn't any other issue or something else that was serious that needed to be looked at, and we could make the discomfort sort of more manageable.” (E) | N/A |
| 1. Covers feedback | Explains that no serious medical problems have been uncovered and that it is safe to gradually resume activities | 1  “… by reading that not all back pain is harmful, like 80% of people got back pain… you can live with it, you can continue being active while you are having the back pain …if you think about it, it’s true, you know, it helps.” (U) |  |
| 1. Shifts focus from pain to function | Rather than struggling with pain, suggests openness to another approach and presents the goal of PACT, to help people function better especially in the areas that are important to them | 7  “The PACT thing is more of a longer term thing so I think that’s key and I think we should carry on. It’s difficult when you're in immediate pain to talk to somebody about the longer term or the medium term, but I think if they get through the initial stages and stuff like that it’s important to carry on.” (F)  “PACT goes in much more depth: how you cope with it, what you do with it, how you can overcome the pain and sort of work round it.” (L)  “But I, I have this voice going though my head all the time, telling me to think of my pain, and, and to put it away, that is very difficult to do. You, when you are in severe pain, you are very tempted to say it to somebody “will you have it then” (N)  “When I’m doing the exercises, she said ‘Take your mind out from the pain and get on with it’. And then I do. The pain won’t go at all but I am trying to take my mind off from it and go and do what I’m supposed to do.” (O)  “Um, I still stoop sometimes but it was almost re-training me or learning to say that you need to work with the pain.” (P)  “So the idea is that you accept your pain by accepting it and owning it, you can move beyond it so that it doesn't impact your life, which is the most important point.” (S) |  |
| 1. Helps patient identify SMARTER goals: | a) Engages patient in identifying core values and setting related goals, b) breaks goals down into small steps, c) records agreed goals in the patient guide | 9  “….If I, for example, [go] walking or exercising for five minutes, next week or four days after, I should set a goal of fifteen minutes.” (C)  “I had a couple of things [in session 1], like, ‘What are my goals?’, ‘What am I trying to achieve through this?’, ‘How can I have a measured structured approach to achieving what my goals are?’” (F)  “I think looking and thinking back now, it was you setting the goals in cooperation with the physio, but not them telling you what you should be doing. They were probing – say, ‘What do you think is your realistic goal?’” (L)  “I think is what PACT has said throughout the sessions I've had is to set goals but them realistic, so that’s what I do.” (N)  “It's structured stuff, instead of just having a general "I feel rotten", it was I can do A B C, and then I'd feel good about it, the fact that I'd just spent 20 minutes in the garden tending the roses, makes you feel good.” (Z) | 3  “I set goals in my life… if you have a work appraisal or an appraisal they've got to be SMART, you know, there’s no point if you can't achieve them, they've got to be realistic, they've got to be timed and they've got to be timelined… achievable.” (F)  “[The physiotherapist] said concentrate on the pleasure you get from those things as part of your goal orientation whereas all my goal orientation has been about work. So now, I'm mixing it up. I'm still keeping the work stuff because I need that to pull me forwards but um, the pleasure ones are there at the end of it.” (S) |
| 1. Addresses barriers to goal attainment | Encourages patient to consider and prepare for potential barriers to goal fulfilment. Implements strategies to promote openness, awareness and engagement, for example mindfulness exercises and action plans in response to potential barriers. | 2  “So, if I were say staying with my mom in [location], that usually was a [barrier] because it wasn't part of my routine or, you know, there's not really the same space available -- and there is usually other things to do.” (E)  “It’s important to actually I’d probably reinforce the first session, if you cannot do something, even if you -, that’s why I said the scores have to be smart, let’s think about alternatives, so things that you'd like to do, alternative way of achieving that goal if you can't do it sort of via Plan A.” (F) |  |
| 1. Teaches “Notice 5 Things” mindfulness exercise*^a^* | Physiotherapist demonstrates Notice 5 Things and reinforces how the patient can use this skill anytime on their own to help when they are struggling with their pain. | 8  “So at first, that was difficult to notice 5 things. And then it was like ‘That's alright, can you notice 3 things?’… So, was it helpful? Yeah. Because it started to help me to focus on learning what mindfulness is and seeing something different.” (P)  “One of the things that PACT taught me was mindfulness, which I had heard of before, but concentrating on other things, becoming aware of your surroundings and then focusing on things that are outside of you, so you're in your environment so that you remove the attention from yourself.” (S) | 4  “I tried notice 5 things. I started looking around the room, and it took me awhile, and then my attention was on something else, and the dentist was doing her job, and I realised it was over.” (A)  “The thinking of five things, possibly. I do it now... I do it when I'm angry as well. I'd do it for loads of things.” (M)  “So now, I'm able to tune everything out and then concentrate on the intake. And then I can actually hear the various components of [the birdsong], the knocking, the shushing… -- and I can break it down. And once you do that, it kind of pulls you away from it, it takes your stress levels down, calms you.” (S) |
| 1. Uses at least one metaphor or tool*^b^* | At any point during the session, at least one metaphor or tool should be referred to. | 1  “…just like the alarm when it rings and then you try to ignore it sometimes it rings it’s not anything.”  (U) |  |
| 1. Provides PACT patient guide*^c^* | Giving the patient knowledge to use outside of the sessions and after treatment | 13  “That'd make me read this up [referring to pact guide] again and say, ‘Oh, let me come to [my] goal and how many goals have I reached’.” (R)  “But having a booklet to read, um, helping you to cope um, and asking you did you refer to the booklet, did you read it? You know, two way, it's a question and answer, which is useful. I remember that happening and I remember the various things in the book.” (L)  “I would never have thought of five things to do… of setting yourself goals and times. I would have just thought you know, you're a British woman, grit your teeth and get on with it [laughter], which is not the best attitude. Um, it explains a lot, I mean, why I still have pain.” (Z) | 8  “…understanding and reading it was very good you know, there was… lots of information, it's really clear explaining what back pain is... -- there's not always a cure for back pain, it's managing pain [referring to pact guide]. And I think that is the main thing that came out.” (L)  “And the good thing is it's something to reach for if you do kind of forget. Because you know, we're humans, we forget. Um, and it can bring it back to the now.” (S) |
| 1. Handshake or verbal agreement*^c^* | Making a public commitment to agreed goals. |  |  |
| 1. Responds positively to patient’s efforts, progress and achievements | Praises patient’s efforts towards goal efforts irrespective of success | 2  “I think the second session served as a reinforcement and it also helped you see that you had done possibly more than you thought you had. I know I felt very proud of myself, quite pleased with myself.” (Z)  “So actually achieving those goals I think um, was hard. So, I knew what my goal was… then I hadn't done it, which was a little bit frustrating. Um, but then what was useful was that I wasn't … reprimanded because I hadn't done it. It was, you know, ‘You’re gonna get through this now, because you haven't done it, but actually you have done these.’ So that was useful to talk about what I had done from what I haven't done.” (P) |  |
| 1. Normalises and empathises with goal challenges | Reminds patient that things do not always go to plan and lots of people have setbacks when trying new ways of doing things |  |  |
| 1. Goal adjustment/ development | Checks the salience of goals and makes adjustments if required, including adjusting steps towards goals. Re-establishes commitment using motivational interviewing techniques if necessary | 2  “I know goals were quite a lot um about the study and the follow-on, how am I going to get on with the goals, have I set realistic targets, and if I haven't set realistic targets, how would I alter them if I couldn't do. I remember those conversations.” (L) |  |
| 1. Integration of self-management approach | Reviews key skills and helps patient identify a support network. Discusses maintenance tools and normalises setbacks | 1  “… discussing that there weren't any kind of setbacks or any other issues in terms of stopping me doing or achieving what I want to achieve. Um, so in that sense it was good. …Everything seemed to be progressing as expected so other than kind of reassurance and just um, getting a little bit of motivation.” (E) |  |
| 1. Discussed integration of goals into daily life | Rehearses new skills, such as mindfulness and shifting focus and explores how these can be extended to other areas of life. Encourages the development of insights and the capacity to self-initiate change |  |  |
| 1. Addresses future challenges, including treatment seeking | Emphasises that the patient will face times when they experience pain or other difficulties, and their natural response will be either that treatment did not work or that they need more. Acknowledges that this is normal and reminds them that they have the skills and resources to carry on without further treatment (e.g. PACT Patient Guide and new skills) |  |  |
| 1. Confident and positive sign off | Positive closure of the therapeutic partnership to help reinforce patient capacity to persist with the tools they have to manage their back pain without needing more health care |  |  |
| Total n =19 | | |  |
